# Globalization of a telepathology network with artificial intelligence applications in Colombia: The GLORIA program study protocol

**DOI:** 10.1016/j.jpi.2024.100394

**Published:** 2024-08-15

**Authors:** Andrés Mosquera-Zamudio, Marcela Gomez-Suarez, John Sprockel, Julian Camilo Riaño-Moreno, Emiel A.M. Janssen, Liron Pantanowitz, Rafael Parra-Medina

**Affiliations:** a Research Institute, Fundación Universitaria de Ciencias de la Salud, Bogotá, Colombia; b Facultad de Medicina, Universitat de València, Av. de Blasco Ibáñez, 15, València, Spain; c Instituto Nacional de Cancerlogía, Bogotá, Colombia; d Faculty of Medicine, Cooperative University of Colombia, Villavicencio, Colombia; e Department of Pathology, Stavanger University Hospital, Stavanger, Norway; f Department of Pathology, University of Pittsburgh Medical Center, Pittsburgh, PA, USA

**Keywords:** Digital pathology, Telepathology, Computational pathology, Telemedicine, Cancer, 0000, 1111

## Abstract

In Colombia, cancer is recognized as a high-cost pathology by the national government and the Colombian High-Cost Disease Fund. As of 2020, the situation is most critical for adult cancer patients, particularly those under public healthcare and residing in remote regions of the country. The highest lag time for a diagnosis was observed for cervical cancer (79.13 days), followed by prostate (77.30 days), and breast cancer (70.25 days). Timely and accurate histopathological reporting plays a vital role in the diagnosis of cancer. In recent years, digital pathology has been globally implemented as a technological tool in two main areas: telepathology (TP) and computational pathology. TP has been shown to improve rapid and timely diagnosis in anatomic pathology by facilitating interaction between general laboratories and specialized pathologists worldwide through information and telecommunication technologies. Computational pathology provides diagnostic and prognostic assistance based on histopathological patterns, molecular, and clinical information, aiding pathologists in making more accurate diagnoses. We present the study protocol of the GLORIA digital pathology network, a pioneering initiative, and national grant-approved program aiming to design and pilot a Colombian digital pathology transformation focused on TP and computational pathology, in response to the general needs of pathology laboratories for diagnosing complex malignant tumors. The study protocol describes the design of a TP network to expand oncopathology services across all Colombian regions. It also describes an artificial intelligence proposal for lung cancer, one of Colombia's most prevalent cancers, and a freely accessible national histopathological image database to facilitate image analysis studies.

## Highlights

• The GLORIA program marks a significant milestone in Latin American Digital Pathology.
• GLORIA integrates public and private healthcare systems to address pathologist shortage.
• The performance evaluation will be through a novel qualitative and quantitative method study.

## Graphical abstract

National Digital Pathology network including Whole Slide Imaging Telepathology System and database for Artificial Intelligence (AI) models. SDC: Satellite Digitization Center COE: Center of excellence.

**Figure:**
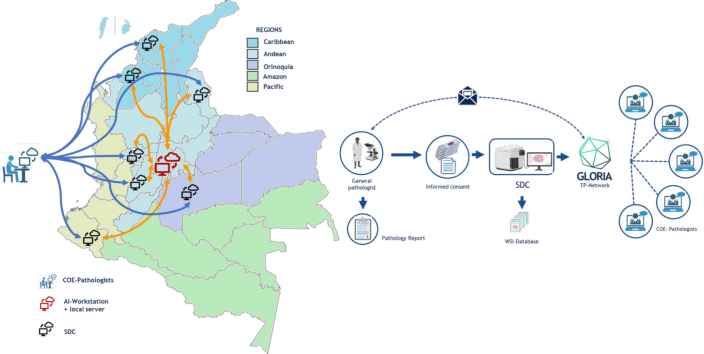

## Introduction

Cancer is the second leading cause of death worldwide. In 2050, approximately 18.5 million people will die from cancer.^1^ Cancer rates and death rates due to cancer are on the rise. The increase can be attributed to factors such as aging and population growth, as well as shifts in the prevalence and distribution of cancer risk factors, all of which are interconnected with the broader dynamics of socioeconomic development.^2^, ^3^, ^4^ These factors contribute to a widening gap in terms of disease burden and access to essential technologies for effective cancer screening, diagnosis, and treatment. Unfortunately, this disparity has led to elevated mortality rates, particularly in low- and middle-income countries.^5^

In Colombia, cancer, classified as a high-cost pathology, faces significant delays in diagnosis and treatment access. The average time it takes to receive a diagnosis can be more than 70 days, with cervix, prostate, and breast cancer having the longest turnaround times (79.13, 77.30, and 70.25 days, respectively). Access to treatment continued to face further delays, with prostate cancer patients waiting an average of 87.66 days, melanoma (81.92 days), and colorectal cancer (61.64 days).^6^

Anatomical pathology, whereby tissue samples from patients are examined under a microscope, plays a vital role in the accurate diagnosis of malignancies.^7^^,^^8^ Apart from rendering a diagnosis, pathology allows the classification and staging of oncological processes. Moreover, ancillary studies provided by pathology, such as biomarker testing and molecular findings, can help personalize patient treatments and clinical prognosis.^9^ Globally, only 26% of low-income countries have pathology services in the public sector, and less than 30% reported that treatment services are available.^10^ In Colombia, according to data from the Colombian Association of Pathology (ASOCOLPAT, for its acronym in Spanish), in 2023, there are approximately 450 pathologists throughout the country, with a rate of 0.88 pathologists per 100 000 inhabitants.^11^ Colombia does not have training programs in pathology subspecialties,^12^ and there is a biased distribution of expert oncopathologists towards the major cities of Bogotá, Medellin, and Cali.^11^ This situation leads not only to decreased access to pathologists with expertise in cancer but also deepens national inequality in healthcare for cancer patients.

In Colombia, the geographical distance of most municipalities from major cities exacerbates oncological diagnostic challenges. While surgical procedures are available at municipal facilities, the lack of local pathology laboratories necessitates transporting patient specimens to reference centers in major cities. This process, often involving patients or specialized logistics, leads to delays, increased costs, and risks such as lost specimens. Even in municipalities with pathology laboratories, there is a scarcity of cancer expertise, complicating cases of complex tumors, and requiring second opinions. The COVID-19 pandemic further strained these systems, although telehealth and digital medicine strategies offered some relief in healthcare continuity. Nevertheless, the field of anatomical pathology lagged in technological advancement, widening the gap in timely cancer diagnosis across Colombian regions.

Digital pathology (DP) has gained much traction ever since whole-slide scanners became commercially available, allowing pathology glass slides to be scanned (digitized).^13^, ^14^, ^15^ Digital slides allow pathologists to remotely view cases on computer monitors, simulating the examination of slides using a traditional light microscope.^16^ The benefits of whole slide imaging (WSI) include workflow efficiency, easy image archive retrieval, telepathology (TP), image analysis, and more recently, artificial intelligence (AI).^14^^,^^17^ Currently, WSI is being used mainly for scientific research and education. While many pathology laboratories around the world have also started to use WSI for routine diagnostic work, widespread adoption is slow due to various barriers, including high cost, regulations, and technophobic pathologists.^17^^,^^18^

We herein present a protocol to develop a national DP system for Colombia to address current challenges in anatomical pathology care called the GLORIA (GLobalización de telepatOlogía en Red con aplicaciones de Inteligencia Artificial; Spanish acronym, or “Globalization of a teLepathology netwOrk with aRtificial Intelligence Applications”; English acronym) program. The aim is to: (1) enable general pathology laboratories throughout the country to utilize this DP infrastructure to improve timely access to expert consultants at specialized oncopathology centers and (2) to create and leverage an image database to stimulate the use of computational pathology by developing, validating, and implementing helpful AI-based tools to augment pathology practices in Colombia.

## Material and methods

Our proposal to create a DP system in Colombia is subdivided into two main projects: (1) Establish a TP network and (2) develop a WSI database to support and stimulate computational pathology studies in addition to the development of AI models in lung cancer.

### Implementation of the TP-network

The project aims to provide general pathology laboratories (regardless of whether they are public or private) with access to expertise for diagnosing complex tumors. This will require creating a national TP network with a hub-and-spoke model where challenging pathology cases encountered by general pathologists at peripheral sites can be triaged and electronically transmitted to subspecialists at regional centers of excellence.

#### Satellite digitization centers (SDC)

**Fig. 1:**
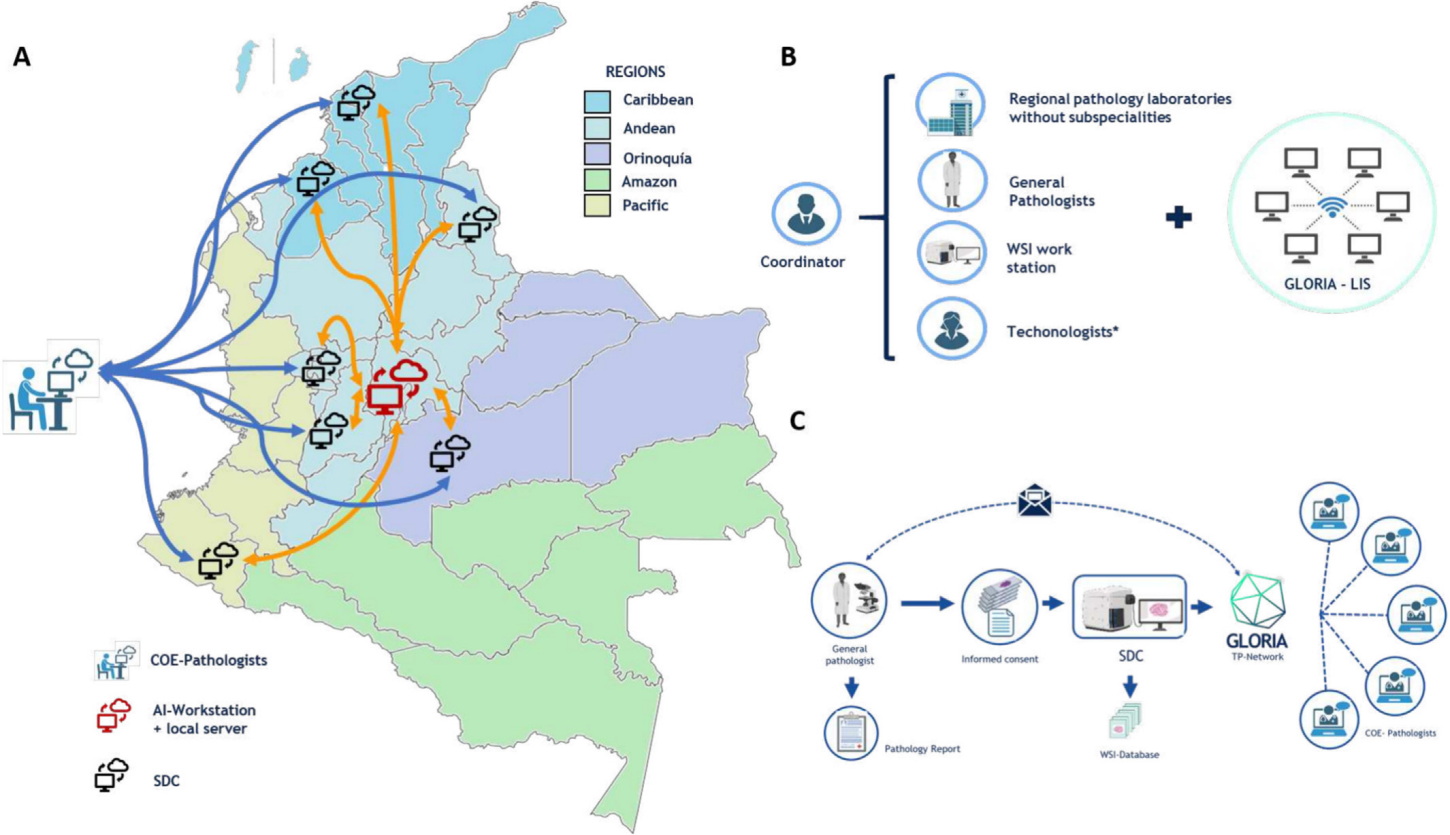
Satellite digitization centers and AI workstation. (A) Distribution of the SDCs in Colombia. (B) Components of the SDC. (C) GLORIA's DP workflow. *Includes IT and cytohistotechology staff. COE: Center of Excellence. LIS: Laboratory information system.SDC: Satellite Digitization Center.

The SDCs will function as TP workstations using a WSI-TP system. These SDCs will be strategically placed in pathology laboratories in different regions of the country (Fig. 1A). Fig. 1B displays the required components for SDC. For deployment of the TP system, there will be an initial period of implementation of technological resources in the SDCs, where hardware such as scanners will be set up in pathology laboratories. This will be followed by validation for clinical use and training of all personnel involved in the TP system and workflow.

#### Teleconsultation workflow

The referring pathologist at the peripheral host location will select relevant pathology slides for digitization to be performed in the SDC. Once scanned and passing the established quality control protocol, these digital slides, along with their metadata (e.g., patient demographics, assigned case number, etc.) can get sent securely for consultation to certified expert oncopathologists in the Colombian Pathology Association according to their field of organ expertise. The images must be accompanied by respective clinical history and if necessary, image annotation (e.g., to indicate the region of interest). The referring pathologist and oncopathologist can communicate through bidirectional means such as teleconferences and simultaneous image viewing to address any need for clarification. After case review, the expert pathologist will formally report their diagnostic opinion to the referring pathologist. The referring pathologist will receive the expert oncopathologist's report to further assist with patient management. There will also be an option to generate teleconsultation with other experts outside Colombia. Fig. 1C presents this proposed TP workflow.

The pathology laboratory that requested a consultation will be responsible for any procedures and logistics necessary to carry out required additional analyses (e.g., perform deeper sections, special stains, etc) if these are available in the general laboratory. Should ancillary analyses be needed that are not available on the menu at a peripheral lab (e.g., molecular testing), additional material (e.g., tissue blocks) can be sent to a core laboratory where such testing is available.

### WSI database for computational pathology studies

The GLORIA program will create a WSI database from all the cases sent for teleconsultation. This database will serve as a collection of images of malignant tumors used to train and validate AI models and for academic purposes.

#### Technical implementation of the WSI database

The GLORIA program utilizes .TIF format images at 40× magnification. Each case will include an average of two WSI, chosen by expert pathologists as the most representative of each case. The metadata system will be stored in a pathology laboratory information system (LIS) with supporting secure data encryption and local technical support. This system will facilitate the necessary metrics to calculate the execution time for each process step, starting from case digitization at each SDC. The elements considered for estimating the storage of the WSI database and WSI in the cloud for teleconsultation are explained in Table 1.

**Table 1:** Storage information for the WSI database.

Element	Quantity
SDC	7
Estimated gigabytes per WSI	3
Months for TP system execution	20
Cases per SDC/month	30
Estimated average number of slides for teleconsultation sharing per case	5
Estimated average number of slides for WSI database per case	2
Gigabytes for total SDCs/month for teleconsultation	3150
Total gigabytes storage/total months of system execution/total SDC for image database	25 200

**Fig. 2:**
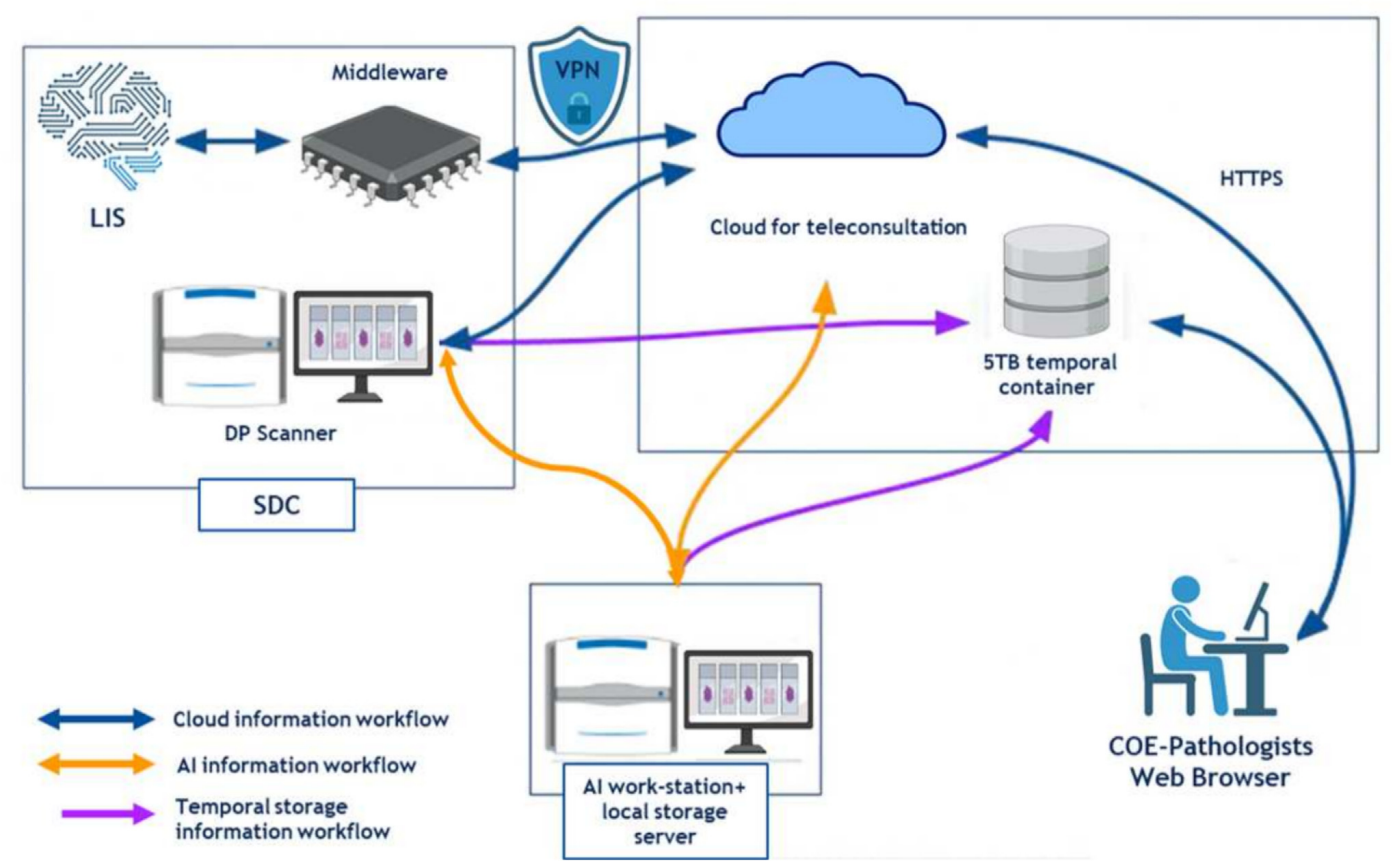
Technological components and workflow.

We will use two storage systems: on-premise with a local server and in the cloud for the case consultation network. The cloud storage capacity of five terabytes will be temporary and used solely for viewing by expert pathologists, with cases not expected to remain longer than necessary. This space will be efficiently managed by reusing it and discarding WSIs not selected for the creation of the WSI database. The cloud system is a closed system that allows image sharing only via streaming, and downloading images is not authorized. This set-up provides greater security to protect the images. The selected images for the WSI database will be stored on a server located at the AI workstation in Bogotá. See Fig. 2.

#### Evaluation of the TP network performance: Mixed methodology integrating quantitative and qualitative approaches

**Fig. 3:**
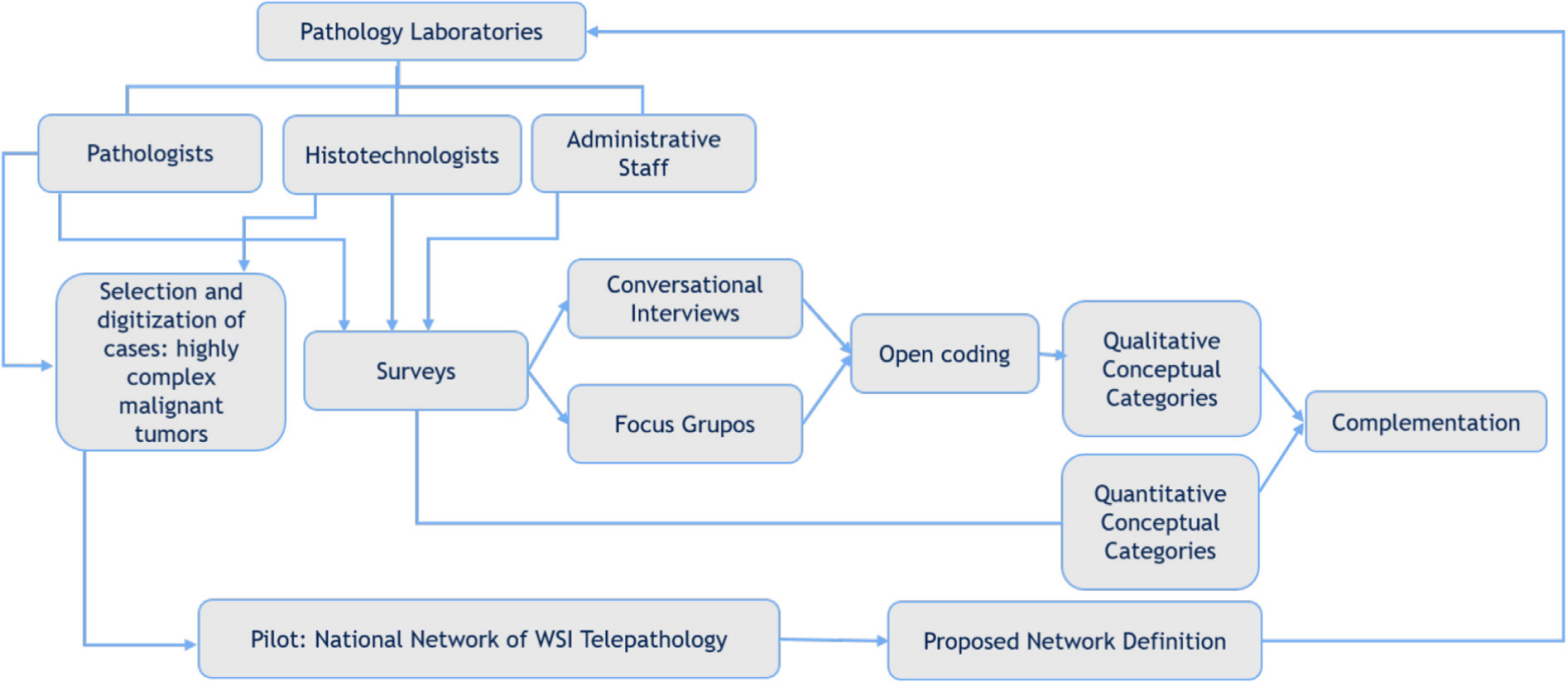
Flowchart of the mixed methods evaluation of the TP-network process.

Using a comprehensive approach that accounts for various factors (such as personnel, pathology facilities, and underlying IT infrastructure), we propose a mixed-method research design where quantitative and qualitative phases will be applied concurrently and in parallel before and after the TP network implementation. The objective is to compare results from these distinct stages and then consolidate them through a methodological complementation process, ensuring that the same selected group is assessed in both phases. (Fig. 3).

#### The quantitative approach

The proposed quantitative methodological approach is designed to describe, in quantitative terms, the perceived needs of general pathology laboratories regarding the diagnostic timeliness for highly complex malignant tumors and to identify which of these tumors frequently present diagnostic challenges. To achieve this, we designed a questionnaire to be implemented throughout the project for each SDC (see Table 2, Table 3).

**Table 2:** Cases distribution of the TP-consultation cases.

Variable	Operational level
Geographic location	Andean
	Caribbean and Insular
	Orinoquia
	Pacific
	Amazonian
Age	Years
Biological sex	Female
	Male
	Other
Types of biopsy	Incisional biopsy
	Excisional biopsy
	Cytology
Anatomical region	Brain
	Head and neck
	Lung
	Mediastinum
	Gastrointestinal
	Breast
	Bone and soft tissue
	Thyroid
	Urogenital
	Gynecological
	Hematolymphoid
	Skin
Response time	Days
Consultation result	Resolved
	Unresolved
Affiliation health system	Public
	Private

**Table 3:** Quantitative survey of SDC staff. * question for pathologists only.

Question	Answer alternatives
What is your level of academic training?	Technologist
	General Pathologist
	Subspecialist Pathologist
How many years of experience do you have in your profession?	Completed years
How many consultations do you perform per month?*	Continuous number
Do you have medical records that include clinical, imaging, and surgical data?*	Yes
	No
In your activity, have you felt the need to be supported by a subspecialist pathologist in high complexity entities?*	Yes
	No
Have you used any telepathology services in your medical practice?	Yes
	No
Are you familiar with the term telepathology?	Yes
	No
In your perception, would a histological image scanner help to improve the diagnostic opportunity for high complexity tumors?	Yes
	No

#### The qualitative approach

Given the complexity of assessing the felt needs of the pathology laboratory staff (pathologists, histotechnologists, and administrative personnel) involved in diagnostic processes that pertain to the implementation of the TP network, a qualitative phase of this study is proposed based on the main guiding question: “What challenges could be resolved by implementing a teleconsultation system in your laboratory?”

Using the interpretative case study methodology as outlined by Yin,^19^ it aims to examine the phenomenon within its real-life context, particularly when the boundaries between the phenomenon and its context are not clearly evident. For the development of the qualitative methodology, techniques such as focus groups and phenomenological conversational interviews will be used. The first ones represent a form of confirmatory research to identify, confirm, or refute preexisting knowledges, attitudes, and practices related to the main question. The phenomenological conversational interviews as proposed by Van Manen involve an unstructured form of interviewing to explore the individual lived experience diving into a “direct description of a particular sit or event as it is lived without offering causal explanations or interpretative generalizations”.^20^ The members of the SDC work teams who agree to participate voluntarily in this phase will be randomly distributed for conversational interviews or focus groups to promote multidisciplinarity. Theoretical sampling will be employed, using the principle of saturation^21^ as the endpoint for the interviews, that is, when the data no longer provide additional information. In the final process, the development of conceptual categories through open coding^22^ is proposed to illustrate, defend, or challenge the theoretical assumptions.

#### Complementation

Complementation involves correlating quantitative and qualitative findings to identify fundamental concepts that facilitate the creation of a national TP network. This multimethod integration strategy is employed in research focusing on a single subject of study, applying both qualitative and quantitative approaches. It generates two distinct perspectives, each providing a unique view of the observed facts. The results, derived from the methods and techniques specific to each approach, are presented in a two-part report that reveals complementary visions.

After results from both phases are gathered, the integration process entails designing a multimethod matrix that identifies concordant, discordant, and isolated quantitative and qualitative findings. The culmination of complementation is a narrative that enriches and is enriched by the findings of each phase, fostering a dialog between methodological perspectives and allowing meta-inferences that form the foundation for the proposed TP network design.

### AI applications: Primary lung carcinoma

The objective of GLORIA's computational pathology project is to create studies with integrative histology–genomic analysis using multiview deep learning models with our Latin-American population.^23^ In collaboration with the Instituto Nacional de Cancerología in Colombia, the first product of AI applications will be a multimodal model focused on identifying and characterizing primary pulmonary carcinomas at various levels. This will include differentiating cancer from normal and inflammatory tissues, identifying specific types and subtypes of cancer, predicting relevant mutations and the PDL1 status, integrating data from WSI, immunohistochemistry, and molecular findings.

For our study, we aim to include cases of normal lung tissue and each major subtype of lung cancer, including small cell, adenocarcinoma, and squamous cell carcinoma. We will also examine a selection of less common lung cancer types along with non-neoplastic lung pathologies such as inflammatory or preneoplastic conditions. In predicting tumor biomarkers important for directed therapy, we plan to assess a range of cases for various significant markers, compared against a set of negative control cases specifically sourced from pulmonary adenocarcinoma.

We will use two sources to include WSIs of lung cancer in a common database: a retrospective and a prospective source. The retrospective source will consist of images from the historical archives of participating hospitals, which also have clinical, laboratory, and diagnostic imaging data, as well as predictive biomarkers for targeted therapy and PD-L1 status. The prospective source will be collected from cases identified on the GLORIA TP program platform.

Cases will be included if they meet the following criteria: individuals over 18 years old, confirmed cases of lung cancer classified as adenocarcinoma according to WHO classification,^24^ and possessing clinical, laboratory, and diagnostic imaging data along with biomarkers for targeted therapy and PD-L1 status. Cases will be excluded if they cannot be assigned a specific subtype due to lack of consensus among participating specialized pathologists or absence of immunohistochemical or molecular markers, or if the clinical or laboratory information is deemed insufficient.

Different configurations of convolutional neural networks (CNNs) will be trained using Keras and TensorFlow libraries in Python. These configurations include end-to-end CNNs, transfer learning for pre-existing models (such as Inception and EfficientNet^25^^,^^26^), and exploring the applicability of generative adversarial networks.^27^ Parameters will be selected and adjusted for each specific problem, with training utilizing batch backpropagation, RMSProp or ADAM optimization, and dropout rates of 20%–30% to prevent overfitting. Image augmentation will be applied to enhance the training dataset.

The WSI will be divided into smaller patches for classification. Various strategies, such as multiple instance learning,^28^ semantic segmentation,^29^ instance segmentation,^30^ streaming CNN,^31^ and recurrent neural networks, will be explored to determine the best diagnostic performance. The results will be visualized through annotations on original images, heat maps, and other options using Matplotlib. The study aims to include at least 50 cases of normal lung tissue, 150 cases of common lung cancer subtypes, and 50 cases of less common lung cancers, as well as 50 cases of non-neoplastic pulmonary pathologies. Although the sample size is not large, this project serves as a prototype model to initiate future studies on neoplasms in the Colombian population. This methodology aims to balance acquiring a diverse and representative dataset and the practical limitations in sample collection. The inclusion of various lung pathologies and cancer subtypes, along with specific cases for biomarker prediction, is intended to enhance the robustness and applicability of the CNN models developed.

### Ethical considerations

This project presents ethical and legal challenges in Colombia. Although the literature on the potential benefits of pathological digitalization and AI seldom addresses these considerations,^32^ we have undertaken a rigorous approach to ensure compliance with regulatory standards. Our project aligns with the Declaration of Helsinki, CIOMS guidelines, and local Colombian regulations, specifically Resolution 8430 of 1993. Due to its nature and methodology, this research has been classified as “risk-free”.

In the project, two groups of participants are identified: on one hand, pathologists, histotechnologists, and administrative staff of pathology laboratories, and the other, patients who will provide histopathological samples for analysis. Two informed consent processes will be implemented to protect their rights and well-being, one for each group. The first group will consent to actively participate in the qualitative and quantitative phases of the project, including group work and surveys. The second group will consent to capturing and using WSI data and metadata. This latter group is offered the option to participate through extended consent, which includes donating their WSI to create an open database for future research. This group also has the opportunity to withdraw their consent voluntarily at any time.

In terms of specific regulations governing the ethical use of WSI in Colombia, although no law directly addresses WSI, such data are deemed “sensitive” owing to their connection with health information. Therefore, this study will comply with the Colombian Data Protection Law (1581 of 2012)^33^ and the Habeas Data Law (1266 of 2008).^34^ It will also adhere to the National Biobank System Law (2287 of 2023).^35^ This legal framework ensures the maintenance of professional secrecy and the protection of the dignity, integrity, and ethnic diversity of participants, as well as the safeguarding of their freedom. Data will not be used for commercial purposes.

From the digitalization to the storage and use of WSI data, the privacy and confidentiality of the participants will be prioritized. Data encryption will be implemented at rest and in transit to protect data. An anonymization process will be applied for laboratory professionals (first group of participants), while the patients' data (second group) will initially undergo pseudonymization. Our pseudoanonymization system aims to adhere to the principles of privacy by design and by default. At the project's outset, a random number generator assigns a random identifier to each participant. This identifier is used for informed consent, structured data, histopathology slides, and digital images. The datasets are handled in separate matrices, with an additional matrix containing personal data exclusively for re-identification by the research team. They are responsible for linking cases to healthcare facilities for diagnosis. Pathologists supporting the network diagnoses will initially work with anonymized data and analyze the slides and clinical information without patient identifiers. AI models will analyze datasets via the assigned number without personal data, ensuring no re-identification. Participants who donate their WSI to the open database will undergo complete anonymization.

The management, protection, and governance of data within this project will be conducted with utmost rigor at every stage. We will securely store the data on both local and cloud servers, which are explicitly designated for this research. Each research team will be granted exclusive access, with individual partitions set-up for every participating institution to ensure controlled and segregated access. Additionally, for the open database, we have planned local storage that allows for on-demand access. Finally, in terms of AI system development, we are committed to adhering to the ethical framework and governance guidelines proposed by the World Health Organization.^36^^,^^37^

The project has been approved by the Fundación Universitaria de Ciencias de la Salud (FUCS) ethics committee in Bogotá D.C., Colombia.

## Discussion

DP has seen significant growth in recent years, mainly in high-income countries, where it has revolutionized telemedicine systems in pathology (TP), enabling faster and more timely diagnoses by facilitating interactions between general pathology laboratories and specialized pathologists worldwide. It is also increasingly used in scientific research and innovation, aligning with automation and digital transformation. By leveraging big data, data mining, and computational algorithms, AI-based clinical decision-support systems have been developed, enhancing precise and efficient diagnostics and transforming pathology practice and education.

However, unlike radiology, the digital transformation in pathology has been slow, with uneven global growth. In recent years, significant progress has been made in high-income regions, while developing countries have seen slower but steady advancements.

In Canada, since 2011, DP has been pivotal in providing histopathology services across a widespread population. It enables more efficient intraoperative consultations, expert opinions, and urgent analysis. By 2014, thousands of slides were scanned, demonstrating a high concordance rate with traditional methods and reducing response times significantly. This approach has been essential in maintaining services in hospitals without pathologists, preventing the need for multiple surgeries, aiding in recruiting surgeons in remote areas, and decreasing professional isolation among pathologists. However, the broader adoption of TP necessitates technological improvements and better coordination.^38^^,^^39^

In the United States, institutions like the Memorial Sloan Kettering Cancer Center have reported notable efficiency gains with DP, including significant reductions in requests for glass slides and substantial cost-savings. Similarly, the Southwestern Medical Center at the University of Texas showed high diagnostic accuracy using telecytology, indicating its effectiveness.^40^^,^^41^

The adoption of DP has also accelerated in response to the COVID-19 pandemic, as seen in Switzerland. A national survey revealed an increased use of DP for primary diagnostics despite challenges like the absence of standard operating procedures and specialized equipment. In the UK, the Leeds Teaching Hospitals NHS Trust has been a pioneer in using DP for primary diagnosis since 2018, underscoring the importance of technical validation and training.^42^

In regions with fewer resources, like Africa, where the ratio of pathologists to the population is extremely low, TP initiatives have been vital. For example, in Benin, training laboratory technicians in preparing virtual slides have significantly improved cancer diagnosis in areas lacking pathologists.^10^

In Asia, retrospective studies, such as the teleconsultation collaboration between the University of Pittsburgh Medical Center and a major private laboratory in China, have highlighted DP's role in enhancing diagnostic accuracy and altering treatment plans.^43^ Additionally, The Chinese National Cloud-Based Telepathology System, operational for over 4 years, effectively managed large volumes of WSI data, showing increased usage, high diagnostic accuracy, reduced turnaround times, and significant cost-savings, demonstrating its potential to alleviate pathologist shortages and reduce patient costs in China.^44^

Latin America, despite facing technological, infrastructural, and economic barriers, has made strides in DP. Countries like Mexico, Colombia, Ecuador, Peru, Brazil, Chile, and Argentina are utilizing DP primarily for teleconsultations and academic purposes, with Colombia leading in scientific publications in this field.^45^

Our network is set to expand across most areas of Colombia, but currently, it does not cover the Amazon region. This is primarily due to the absence of laboratory services and surgical facilities in the area. As a result, many residents need to travel to major cities like Bogota for these procedures. We plan to address this issue in future stages of the GLORIA network's expansion.

It is important to consider whether the use and access to expert pathology second opinions will enhance the knowledge base of local and regional pathologists. Given their direct exposure to specialists, it is conceivable that there will be a significant transfer of knowledge between the parties involved, leading to an improvement in local expertise and diagnostic capabilities. Additionally, a key focus will be the adaptation of DP across all human resources involved. Our network will prioritize comprehensive training in DP techniques to improve diagnostic standards and medical education.

The TP network cannot directly address the shortage of pathologists in Colombia. However, the network aims to significantly enhance the diagnostic process by enabling non-expert pathologists to quickly consult with experts. This direct and rapid access to specialized knowledge will improve the accuracy and quality of diagnoses. While the overall number of pathologists remains a challenge, the efficiency and effectiveness of their work will be greatly enhanced through TP, leading to better patient outcomes and potentially reducing the time taken for diagnoses. Without access to expert consultation, non-expert pathologists may take considerably longer to diagnose cases, potentially leading to delays in treatment and less accurate diagnoses. The successful implementation of GLORIA faces several challenges, including financial and technological constraints like insufficient electricity and telecommunication infrastructure. A significant hurdle is the requirement for stable, high-speed internet connections. Unfortunately, some regions in Colombia have limited internet capacity, leading to potential delays in image access and issues with internet service interruptions, which can hinder the TP workflow. To mitigate these challenges, we have ensured that the SDCs have the necessary electrical and internet provisions to function optimally, aiming to minimize system inefficiencies and maintain smooth TP workflows. Additionally, there is a risk of resistance from patients and staff within the TP network to accept informed consent, participate in surveys, and adapt to the new system, posing challenges to the sustainability of teleconsultation services.

The GLORIA project is set to deliver impactful outcomes in several key areas. In knowledge generation, we expect to publish high-impact journal articles and a book, showcasing significant academic contributions. The project will also focus on developing talent by engaging medical specialty students and young researchers, offering them a platform for growth in their fields.

For wider knowledge sharing, GLORIA will present papers at conferences, join international networks, organize events, and conduct various educational workshops. These efforts aim to spread knowledge and foster collaborations.

Additionally, GLORIA aims to serve as a model for similar projects in Latin-American countries and low-resource regions, emphasizing not only scientific advancement but also the democratization of knowledge and capacity building in less privileged areas. This project represents a commitment to global collaboration and community upliftment through science and education.

## Conclusions

In conclusion, the GLORIA program marks a significant milestone in Latin-American medical technology, being the first nationwide DP system integrating both public and private anatomic pathology laboratories. This groundbreaking initiative not only aims to optimize the pathology workflow and expedite the diagnosis of complex neoplastic diseases but also takes a novel approach in understanding the specific needs of pathology laboratory staff through a comprehensive mixed-method study. This will aid in the smooth transition to the TP-WSI system and contribute to the cultivation of social knowledge for its widespread use. Furthermore, a key component of this program will be the establishment of a WSI database specifically tailored to the Latin-American population, setting a new standard for pathology practices in the region.

## Declaration of Competing Interest

The authors declare that they have no known competing financial interests or personal relationships that could have appeared to influence the work reported in this paper.
